# Supplementary figures and images for: Experimental nerve block study on painful withdrawal reflex responses in humans
Source: PLoS One. 2024 Aug 16;19(8):e0309048. doi: 10.1371/journal.pone.0309048 (PMC11329128; doi:10.1371/journal.pone.0309048)

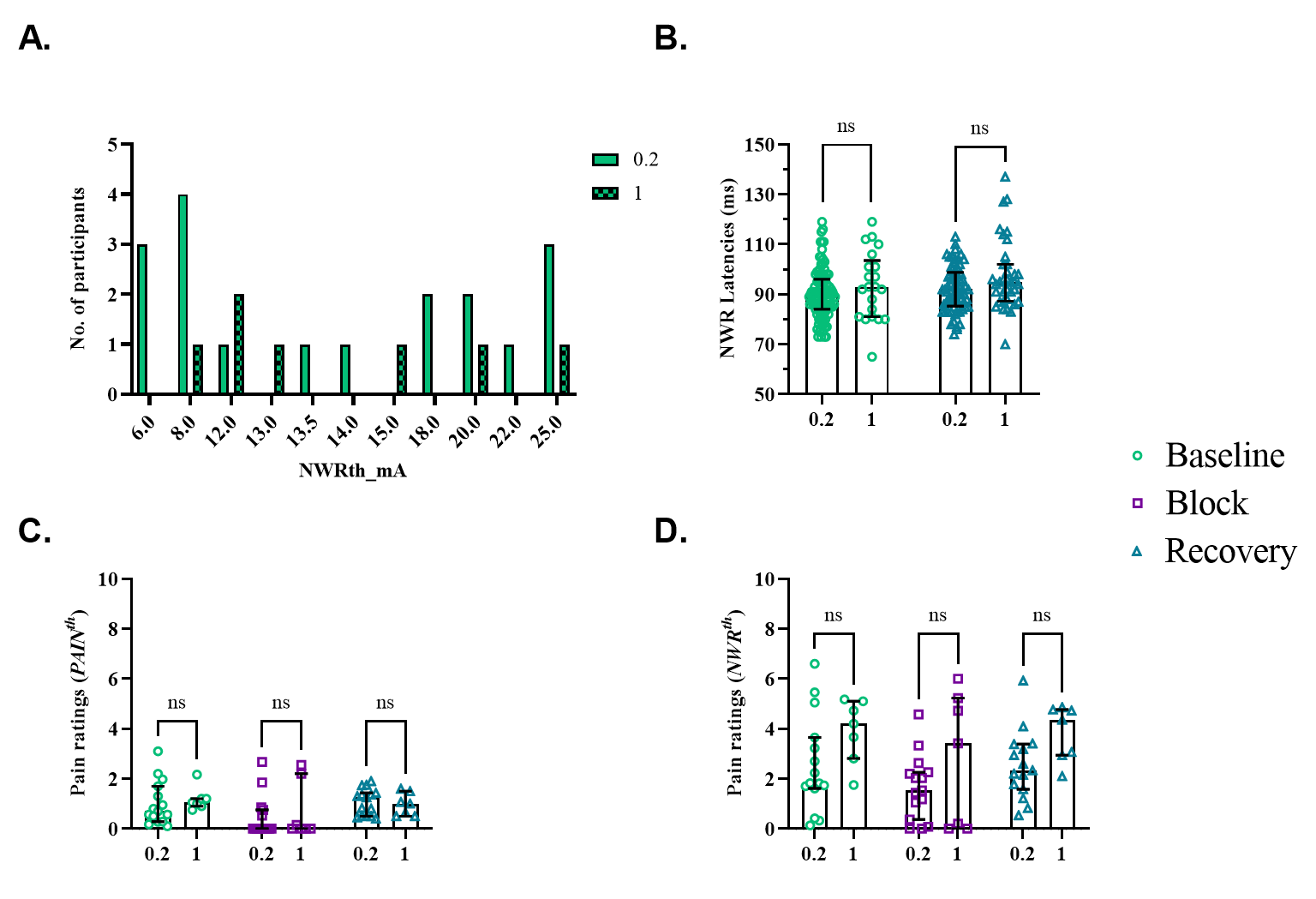

Supplement: S1 Fig — A. Distribution of NWRth separated by pulse duration. B. NWR latencies separated by pulse duration. C-D. Pain ratings at PAINth and NWRth separated by pulse duration across conditions. (TIF) [file pone.0309048.s001.tif]

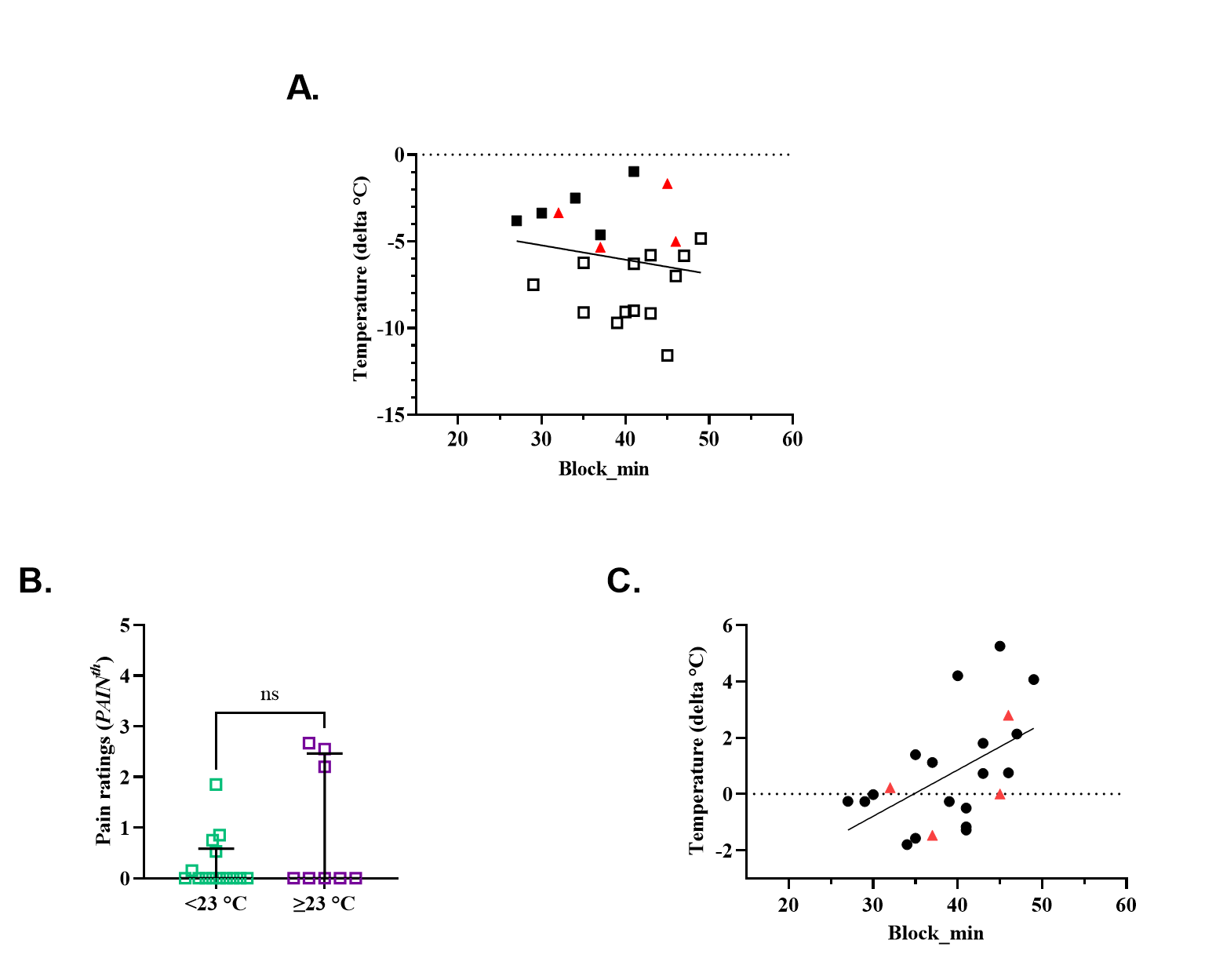

Supplement: S2 Fig — A. Comparison between the duration of nerve block and change in cooling sensitivity. B. Pain ratings at PAINth between subjects with CDT below or above 23°C. C. Comparison between the duration of nerve block and change in warming sensitivity. (TIF) [file pone.0309048.s002.tif]

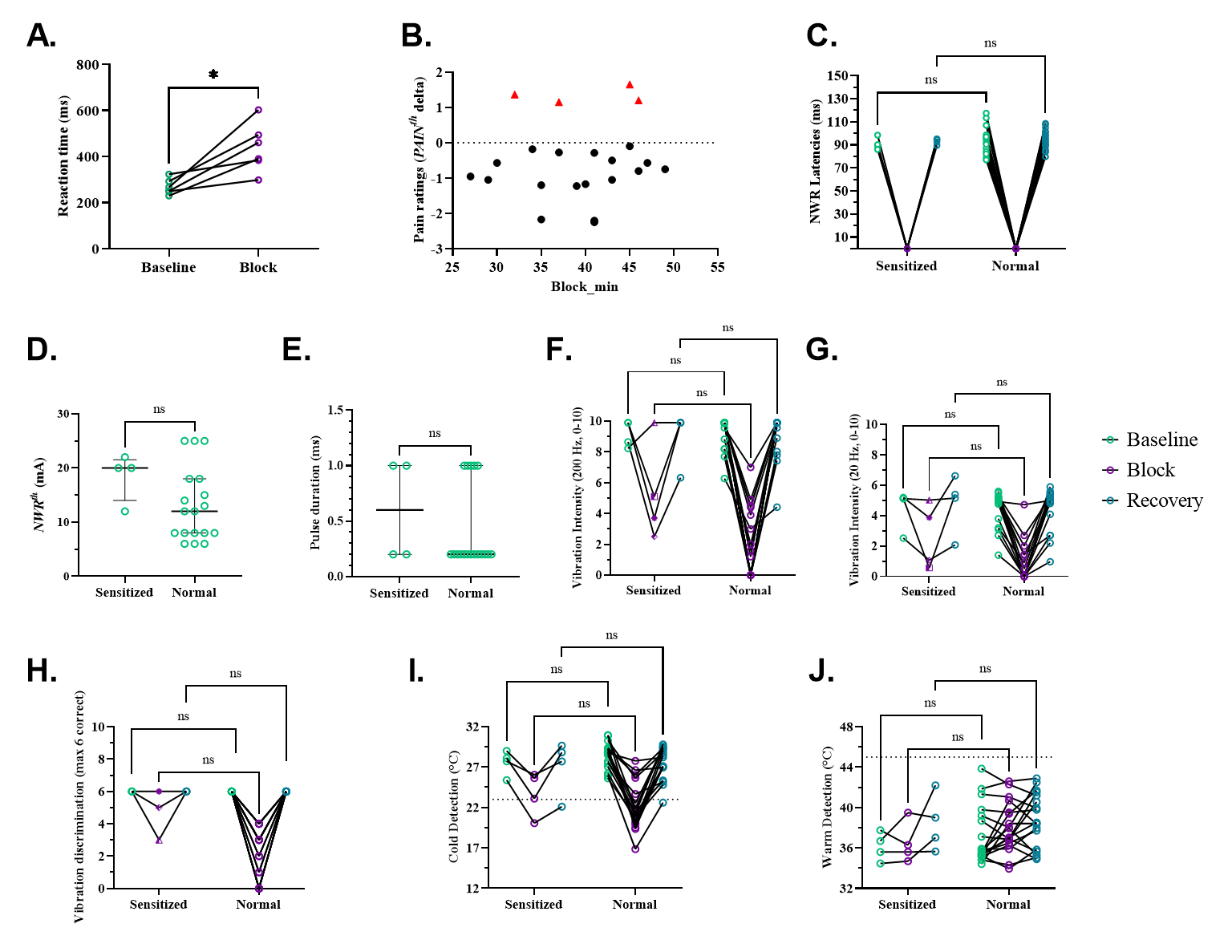

Supplement: S3 Fig — A. Reaction time increased during nerve block. B. Duration of nerve block. C-E. Comparison of reflex latencies, NWRth and pulse duration between sensitized and normal subjects. F-H. Comparison of performance on vibratory tests during the block between sensitized and normal subjects. I-J. Comparison of performance on thermal tests during the block between sensitized and normal subjects. (TIF) [file pone.0309048.s003.tif]

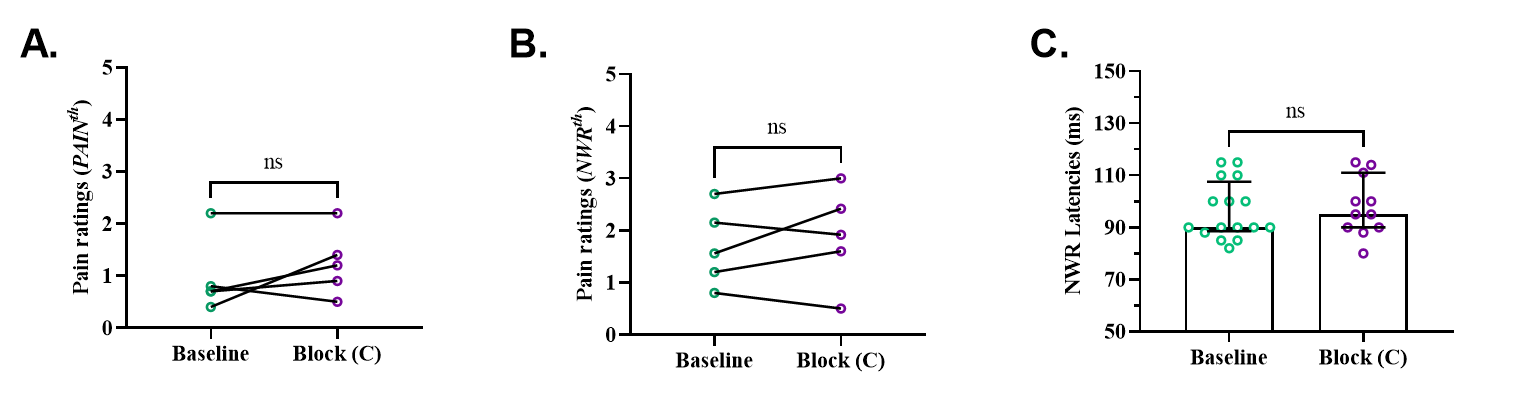

Supplement: S4 Fig — A. Pain ratings at PAINth on the intact test site with a nerve block applied to the contralateral leg. B. Pain ratings at NWRth on the intact test site with a nerve block applied to the contralateral leg. C. NWR latencies on the intact test site with a nerve block applied to the contralateral leg. (TIF) [file pone.0309048.s004.tif]
