# Supplementary material for: Experimental nerve block study on painful withdrawal reflex responses in humans
Source: PLoS One. 2024 Aug 16;19(8):e0309048. doi: 10.1371/journal.pone.0309048 (PMC11329128; doi:10.1371/journal.pone.0309048)
Supplement: Attachment — Submitted filename: Rebuttal letter_PlosOne.docx [file pone.0309048.s006.docx]

We would like to thank the reviewers for their helpful and supportive feedback.

The following text provides a point-by-point response to each of the questions raised. Any quotations from the manuscript are shown in *italics*, and specific changes or additions are shown in red. Supporting text from the submitted manuscript has been identified and reproduced where appropriate. We hope these changes address your questions and look forward to hearing from you about our submission.

For ease of reading, some abbreviations are listed below:

- NWR: Nociceptive Withdrawal Reflex
- NWR^th^: Nociceptive Withdrawal Reflex Threshold
- PAIN^th^: Pain Threshold

**Reviewer 1#**

1. Is it necessary to increase the number of participants more to obtain clearer results? Was there any confounding between age/gender and experimental results?

Detailed response:

In this study, our main finding was the complete abolition of the NWR response, observed in each participant. In the absence of pre-existing data for direct comparison, our initial power calculations were based on pain ratings, from a pilot study (n=7), at PAIN^th^ evoked by trans-cutaneous electrical stimulation (c.f., intradermal stimulation in this study), *where we observed an effect size (f) of 0.255. We then used a 1-β error probability (power) of 0.80, and α error probability of 0.05, which gave a total sample size of 27. Post hoc power analysis, based on f(0.27), α(0.05), and a sample size of 25, gave a power of 82.6. Sample size and power calculations were performed in G* Power (open software, v3.1.9.7)*, which indicated enough power.

A correlation matrix showed no significant correlations between age, sex, NWR^th^ and PAIN^th^ (with the lowest p value = 0.151 between age and NWR^th^). NWR^th^ is not known to be affected by age or sex (Neziri, Andersen et al. 2010), and although PAIN^th^ has been reported to increase with age (Neri and Agazzani 1984), all participants in this study belonged to a relatively narrow age bracket (median age: 23.5 years, range: 18-39 years).

Corresponding changes to the manuscript:

- Lines 84-85: *Twenty-five healthy participants (17 males and 8 females), aged between 18 and 39 years (median age: 23.5 years), took part in this study.*
- Lines 211-212: *Detection, pain, or reflex thresholds did not show any correlation with the age or sex of participants.*

2. The aim of this (or future) work could be better defined, for instance to study interplay between Aβ and Aδ nerve activity under various (patho)physiological conditions.

Detailed response:

While NWR has been extensively studied, including in various pathological conditions (Sandrini, Arrigo et al. 1993, Andersen, Finnerup et al. 2004, Banic, Petersen-Felix et al. 2004, Courtney, Lewek et al. 2009, Biurrun Manresa, Neziri et al. 2011, Ydrefors, Karlsson et al. 2020), the contribution of specific A-fiber classes remains poorly characterized. This is likely due to the difficulties inherent in isolating individual contributions within an intact system when using electrical stimulation alone, prompting us to use preferential nerve conduction blocks in the current study. Moving forward, studying patients with selective large or small fiber neuropathies may provide greater insights into the roles of specific A-fiber classes.

Corresponding changes to the manuscript:

- Lines 331-332: *Moving forward, studying NWR in patients with selective large- or small-fiber deafferentation may provide valuable insights into distinct peripheral afferent contributions.*

3. The authors in the discussion section reported that “Intradermal electrodes, as used in the current study, are likely to stimulate the terminal branches of cutaneous afferents rather than the nerve endings themselves, resulting in a less synchronized afferent volley, perhaps resembling a more natural stimulus compared to surface electrodes”. I think it would be helpful to compare and contrast the differences due to these different approaches, as well as to discuss the feasibility of this latter approach.

Changes to the manuscript:

- Lines 357-361: *While one study found no difference between the two methods (Meinck, Piesiur-Strehlow et al. 1981), another study using both needle and surface electrodes noted the absence of the early component of the reflex response (RII) during surface stimulation (de Willer 1977). However, when comparing the methods, in that study, the sural nerve was stimulated perineurally, whereas in the current study, we stimulated the reflex receptive field of the tibialis anterior muscle by inserting needle electrodes under the skin of the foot sole.*

The following text was removed from the manuscript to avoid confusion:

- *Intradermal electrodes, as used in the current study, are likely to stimulate the terminal branches of cutaneous afferents rather than the nerve endings themselves, resulting in a less synchronized afferent volley, perhaps resembling a more natural stimulus compared to surface electrodes* *(Shahani and Young 1971).*

**Reviewer #2**

Major comments:

Methods

line 27 (and 203)

It is mentioned that during the intact condition 198 NWR responses were recorded. Does this mean 9 per participant (as only 22 showed reflex responses)? How many elicitations were applied per participant? And how many were successful in obtaining a NWR response? I.e. what was the response rate? How many elicitations were applied in the other two conditions? This relevant information seems to be completely missing.

Detailed response:

In our study, a distinction was made between “NWR responses”, which refers to all instances where an NWR was elicited, and “NWR thresholds”, which refers only to NWR responses at a specific current (mA). *NWR thresholds (NWR^th^) were determined based on at least two successful trials at the same current*. All participants had an NWR response, and each participant had a corresponding NWR^th^.

Corresponding changes to the manuscript:

- Lines 204-207: *Under intact (baseline) conditions, 198 NWR responses were obtained from 22 out of 25 participants, ranging from 4 to 16 responses per participant. A reflex threshold (NWR^th^) was established for all 22 participants. Three participants were excluded because the full experiment could not be performed, resulting in insufficient data.*
- Lines 216-221: *During the block condition, the NWR^th^ was tested at least 3 times using each participant's individual pre-block current. Further, suprathreshold intensities (up to twice the pre-block current) were tested during the block condition. This was to make sure that any effect on the NWR during the block was not merely due to a shift in NWR^th^, but rather indicated a completion abolition of the NWR. In the recovery condition, the NWR was considered recovered if it was elicited at least once at the pre-block current threshold.*

lines 104-108

Why was a pulse duration of 0.2ms chosen? It seems extremely short to elicit reflex responses and is not common in NWR studies. For instance, reference 16 uses the typical 1ms duration (see also reference 7). Using short pulse durations increases the risk of the stimulation not being perceived at low stimulation currents. The statement in the methods that, in cases where no reflexes could be elicited "a longer pulse duration of 1ms was used", acknowledges this issue (which affected 7 out of 22 participants, see lines 210-211). Therefore, please justify the chosen setting of 0.2ms or provide pertinent references.

Detailed response:

Although 1 ms duration is often used, shorter pulse durations have also been favored (Meinck, Kuster et al. 1985). We used closely spaced (5-mm) intradermal electrodes to apply *short-duration electrical pulses (0.2 ms) to preferentially activate larger-diameter fibers, based on the strength-duration relationship for electric excitation of myelinated axons (Hill 1936, Szlavik and de Bruin 1999, Wesselink, Holsheimer et al. 1999).*

Participants clearly perceived the stimulus. *The current intensity required to reach DET^th^ was the lowest, followed by PAIN^th^, and finally NWR^th^.* *All reflex thresholds occurred in response to a painful stimulus, with pain intensity ratings ranging from 0.3 to 6 on a VAS of 0-10.*

Corresponding changes to the manuscript:

- Lines 102-105: *In microneurography, short pulse durations have been shown to evoke a pain percept when Aβ nociceptors are selectively activated (Nagi, Marshall et al. 2019). Our approach, using needle electrodes, aimed to minimize current spread. Therefore, we chose the shortest pulse duration at which an NWR could still be elicited.*
- Lines 365-368: *We used short-duration electrical pulses (0.2 ms) to preferentially activate larger-diameter fibers, based on the strength-duration relationship for electric excitation of myelinated axons (Hill 1936, Szlavik and de Bruin 1999, Wesselink, Holsheimer et al. 1999) and to reduce the potential for irregular repetitive firing from primary afferent fibers in response to prolonged stimulation (Meinck, Kuster et al. 1985).*

Was it checked whether the choice of pulse duration and the obtained reflex threshold were significantly associated or not? Given the huge spread of obtained thresholds (visible in Fig. 1-B) one might want to make sure that this is not just due to the chosen pulse duration. Lines 211-213 only refer to potential differences in latencies but does not mention the threshold values, whose distribution is shown in Fig. S1-A.

Response:

No difference was found between NWR^th^ at 0.2 and 1 ms.

Corresponding changes to Supplementary 1:

- (NWR^th^ 0.2: 12.0 (12.0), NWR^th^ 1.0: 13 (8.0), p = 0.500, U = 42.5, n = 22, Mann-Whitney test).

Corresponding changes to the manuscript:

- Lines 229-231: *In a subset of participants (n = 7), the reflex could only be evoked using a 1-ms pulse duration. However, there were no statistical differences in NWR^th^, reflex latencies, or pain ratings based on pulse duration, hence the data were combined (Fig S1A-D).*

line 128

Why was the reflex window set to 50-150ms post stimulus? According to reference 7, typical latencies for RII are between 40-60ms post stimulus. And according to 9, "a natural separation point between RII and RIII reflexes would be around 60ms".

Detailed response:

The choice of reflex time analysis window depends on the focus of the study. Due to a clear baseline, and small stimulus artefacts, we were able to detect NWR responses within milliseconds of stimulus onset, although the shortest latency observed was 65 ms. The short-latency responses reported by Hugon (Hugon 1973) were following non-nociceptive stimulation.

Corresponding changes to the manuscript:

- Lines 131-134: *Following individual inspection of EMG recordings, a time analysis widow of 50-150ms was adopted. Z scores (> 1) were calculated as the difference between peak amplitude (50-150 ms post-stimulus onset) and the mean baseline amplitude (-0.15 to 0 ms relative to the stimulus onset), divided by the standard deviation of baseline EMG activity.*
- Lines 345-351: *The rapid return to baseline, following stimulation, allowed clear visual inspection of EMG recordings for short-latency responses. Since no NWR responses occurred prior to 50 ms, an analysis window of 50-150 ms was adopted to include both short and long latency responses.* *Upon examining data from all individual reflex trials, only 9 out of 198 reflex responses (4.5%) were perceived as non-painful. These non-painful reflexes did not have the shortest latencies. The absence of pain in these instances might be due to a momentary shift in the participants’ attention, as they never rated two consecutive reflex responses as non-painful.*

Results

lines 273-275

Were these four participants different from the rest w.r.t. to NWR threshold stimulation current? It is mentioned in lines 381-382 that NWR responses did not differ between these four and the rest. But no mention is made about the NWR threshold.

Changes to the manuscript:

- Lines 290-293: *Further, these four participants were not different from the others when comparing reflex latencies, pain ratings at PAIN^th^ or NWR^th^, vibration sensibility, or temperature thresholds. They also did not differ based on NWR^th^ or pulse duration (Fig S3C-J).*

Following figures have been added to Supplementary 3 (D and E):

Corresponding changes to Supplementary 3:

- ***C-E. Comparison of reflex latencies, NWR^th^ and pulse duration between sensitized and normal subjects.*** *To simplify, subjects with an increased PAIN^th^ during the block are referred to as ‘sensitized’ while subjects who did not have an increase are referred to as ‘normal’. NWR latencies did not differ between sensitized (n=4) and normal (n=18) subjects (Sensitized: baseline 89.6 (9.7) ms, block 0.0 (0.0) ms, recovery: 93.6 (4.8) ms****;*** *Normal****:*** *baseline 91.8 (11.0) ms, block 0.0 (0.0) ms, recovery 93.9 (12.7) ms, f(2) = 42.79, p < 0.001, post hoc test: p(baseline & recovery) > 0.999, n = 22, Kruskal-Wallis test).* *NWR^th^* and pulse durations were not different in the sensitized subjects compared to normal subjects (*NWR^th^* Sensitized: 12 (10.0), Normal: 20 (7.5), p = 0.137, U = 18, n = 22, Mann-Whitney test) (Pulse duration Sensitized: 0.6 (0.8), Normal: 0.2 (0.8), p = 0.565, U = 28, n = 22, Mann-Whitney test).

And in lines 205-207

it is mentioned that 90ms was seen as the cutoff for RII reflexes. So, why and how was the 50-150ms window assessed?

Changes to the manuscript:

- Lines 224-225: *Out of these, 80 (40.4%) NWR responses had latencies under 90 ms, a cutoff defining onset of RIII reflexes, as used in prior studies (e.g. Sandrini, Arrigo et al. 1993, Ruscheweyh, Kreusch et al. 2011, Ydrefors, Karlsson et al. 2020).*

Discussion

lines 329-331 (and 109-111)

It has been observed that attention and distraction are associated to reflex responses (see Bjerre L. et al., "Dynamic tuning of human withdrawal reflex receptive fields during cognitive attention and distraction tasks", EJP 2011). Was attention or distraction of the participants, especially during reflex elicitations, controlled for in a systematic way? If so, please state how this was done. If not, please justify or elaborate.

Detailed response:

Cognitive influences on reflexes have been investigated in other studies, including the one cited by the reviewer. Our focus here, however, was on the peripheral afferent contributions; nonetheless, to the extent possible within the scope of this work, some considerations were made. For instance, *interstimulus intervals were varied from trial to trial (at least >6 s) to prevent habituation and/or cognitive suppression of the reflex response.* Also, *to avoid visual and auditory cues, a partition was placed between the participant and the experimenter, and a silent mouse was used to trigger the stimuli.* It has indeed been shown that habituation can inhibit reflex responses (Dimitrijevic, Faganel et al. 1972), while anticipation can facilitate them (Willer 1980).

Corresponding changes to the manuscript:

- Lines 428-436: *NWR responses can also be modulated by cognitive factors, such as stress, anticipation, distraction, and emotions (Willer, Boureau et al. 1979, Willer 1980, Sandrini, Milanov et al. 2000, Ruscheweyh, Kreusch et al. 2011, Toledo, Vore et al. 2024). Although these aspects have been explored in other studies, they were beyond the scope of the current work. Nonetheless, certain measures were implemented; for instance, inter-stimulus intervals were varied between trials to prevent habituation, and care was taken to eliminate auditory and visual cues during testing. Our control experiment, involving a pressure cuff on the contralateral leg, further supports that the abolition of reflex and pain was primarily due to the blockade of peripheral input rather than top-down modulation.*

We have also changed the order of paragraphs in the Discussion to improve flow. The paragraph discussing cognitive factors now directly follows the one on spinal gating (diffuse noxious inhibitory control).

Minor comments:

Abstract

line 29

misplaced "was"; it should read "...the NWR magnitude was..."

Changes to the manuscript:

- Lines 29-30: *The current required to elicit the NWR was higher than the perceptual pain threshold, indicating that NWR did not occur before pain was felt.*

Methods:

line 115

"Current intensities were slowly increased in increments of 1-3 mA"

Was this systematically varied between participants? Why were different increments used? Please briefly explain this point.

Detailed response:

The use of different increments is a common practice in threshold testing, even more so when stimulations are manually controlled, like in the current study.

Corresponding changes to the manuscript:

- Lines 115-116: *Current was slowly increased and decreased in increments of 1-3 mA until the first (nonpainful) sensation was reported.*

lines 123-124

Participants had to fill in the short form of the McGill Pain Questionnaire a total of six times. Does this mean the procedure of threshold determination was performed six times in total (thrice for pain threshold and thrice for reflex threshold)?

Corresponding changes to the manuscript:

- Line 119: *PAIN^th^ and NWR^th^ were established during baseline.*
- Lines 125-127: *The pain questionnaire was administered a total of six times, once for each condition (baseline, block, recovery) at pre-block PAIN^th^, and once for each condition at pre-block NWR^th^.*

line 129

Having a baseline window that extends up to the very moment of stimulus onset can prov difficult because of artefacts from the stimulus. Was this checked during data analysis?

Detailed response:

Our earliest NWR response occurred 15 ms after the start of the time analysis window (starting at 50 ms, with our earliest NWR response observed at 65 ms after stimulus onset). Unlike with surface electrodes, where the baseline can be sinusoidal and have large stimulus artifacts, especially with high currents and long pulse durations, our setup using needle electrodes and short pulse durations produced a non-sinusoidal baseline and allowed clear visual inspection of EMG responses immediately after stimulation.

Please see previous response regarding Methods, line 128 (and the corresponding changes made in the manuscript, lines 345-351).

again in line 165

What does "at leas 3 times" mean?

See response to Methods, line 27 (and changes made to manuscript, line 216-221).

Corresponding changes to the manuscript:

- Lines 167-169: *In the optimal condition where the vibration sense was blocked while temperature senses remained, perceptual responses were tested at least 3 times at PAIN^th^ and NWR^th^ intensities.*
- Lines 177-178: *Upon release of the nerve block, vibratory and thermal sensibilities were monitored, and upon recovery to pre-block levels (typically within 20-30 min), NWR^th^ and PAIN^th^ were measured again.*
- Lines 262-263: *During the nerve block, when the reflex was tested at NWR^th^, all responses at their pre-block NWR^th^ were abolished (Fig. 2F).*

line 136 (and 173-174)

Was it checked whether participants felt pain from the ischemic nerve block, e.g. at the ankle, and how this might have influenced the results?

Detailed response:

*To confirm that any effect of the nerve block on pain and reflex responses was due to the blockade of peripheral A-fiber inputs rather than top-down factors (e.g., Diffuse Noxious Inhibitory Control), we ran control experiments with the ischemic cuff applied to the contralateral (left) leg in five participants.* We found that pain and the NWR persisted ipsilaterally, indicating that the diminution of pain and the complete abolition of NWR resulted from the peripheral nerve block, rather than top-down effects.

Corresponding changes to the manuscript:

- Lines 424-426: *No participant reported pain from the nerve block, and although some found the pressure cuff uncomfortable, it was transient, and the experiments continued to completion.*

Fig. S3-A

Maybe indicate "Reaction Time (msec)" instead of just "Time (ms)" on the vertical axis.

The change was made to the y-axis in Supplementary figure S3-A:

General
The wording "current intensity" should not be used when referring to electricity because it simply is unscientific. An electrical current is measured in Ampère and refers to the local change in electrical charge per unit of time whereas an intensity refers to a power measurement (for instance in Watt) and -in electrodynamics- would be proportional to the square of the current. Thus, it is wrong to talk about "intensity" in the present context.

Detailed response:

We have replaced the term “current intensity” throughout the article with “current” when referring to the electrical stimulus.

References

Andersen, O. K., N. B. Finnerup, E. G. Spaich, T. S. Jensen and L. Arendt-Nielsen (2004). "Expansion of nociceptive withdrawal reflex receptive fields in spinal cord injured humans." Clin Neurophysiol **115**(12): 2798-2810.

Banic, B., S. Petersen-Felix, O. K. Andersen, B. P. Radanov, P. M. Villiger, L. Arendt-Nielsen and M. Curatolo (2004). "Evidence for spinal cord hypersensitivity in chronic pain after whiplash injury and in fibromyalgia." Pain **107**(1-2): 7-15.

Biurrun Manresa, J. A., A. Y. Neziri, M. Curatolo, L. Arendt-Nielsen and O. K. Andersen (2011). "Test-retest reliability of the nociceptive withdrawal reflex and electrical pain thresholds after single and repeated stimulation in patients with chronic low back pain." Eur J Appl Physiol **111**(1): 83-92.

Courtney, C. A., M. D. Lewek, P. O. Witte, S. J. Chmell and T. G. Hornby (2009). "Heightened flexor withdrawal responses in subjects with knee osteoarthritis." J Pain **10**(12): 1242-1249.

de Willer, J. C. (1977). "Comparative study of perceived pain and nociceptive flexion reflex in man." Pain **3**(1): 69-80.

Dimitrijevic, M. R., J. Faganel, M. Gregoric, P. W. Nathan and J. K. Trontelj (1972). "Habituation: effects of regular and stochastic stimulation." J Neurol Neurosurg Psychiatry **35**(2): 234-242.

Hill, A. V. (1936). "The Strength-Duration Relation for Electric Excitation of Medullated Nerve." Proceedings of the Royal Society of London. Series B, Biological Sciences **119**(815): 440-453.

Hugon, M. (1973). Exteroceptive Reflexes to Stimulation of the Sural Nerve in Normal Man. Human Reflexes, Pathophysiology of Motor Systems, Methodology of Human Reflexes. J. E. Desmedt. Brussels, Karger. **3:** 713-729.

Meinck, H. M., S. Kuster, R. Benecke and B. Conrad (1985). "The flexor reflex--influence of stimulus parameters on the reflex response." Electroencephalogr Clin Neurophysiol **61**(4): 287-298.

Meinck, H. M., B. Piesiur-Strehlow and W. Koehler (1981). "Some principles of flexor reflex generation in human leg muscles." Electroencephalogr Clin Neurophysiol **52**(2): 140-150.

Nagi, S. S., A. G. Marshall, A. Makdani, E. Jarocka, J. Liljencrantz, M. Ridderstrom, S. Shaikh, F. O'Neill, D. Saade, S. Donkervoort, A. R. Foley, J. Minde, M. Trulsson, J. Cole, C. G. Bonnemann, A. T. Chesler, M. C. Bushnell, F. McGlone and H. Olausson (2019). "An ultrafast system for signaling mechanical pain in human skin." Sci Adv **5**(7): eaaw1297.

Neri, M. and E. Agazzani (1984). "Aging and right-left asymmetry in experimental pain measurement." Pain **19**(1): 43-48.

Neziri, A. Y., O. K. Andersen, S. Petersen-Felix, B. Radanov, A. H. Dickenson, P. Scaramozzino, L. Arendt-Nielsen and M. Curatolo (2010). "The nociceptive withdrawal reflex: normative values of thresholds and reflex receptive fields." Eur J Pain **14**(2): 134-141.

Ruscheweyh, R., A. Kreusch, C. Albers, J. Sommer and M. Marziniak (2011). "The effect of distraction strategies on pain perception and the nociceptive flexor reflex (RIII reflex)." Pain **152**(11): 2662-2671.

Sandrini, G., A. Arrigo, G. Bono and G. Nappi (1993). "The nociceptive flexion reflex as a tool for exploring pain control systems in headache and other pain syndromes." Cephalalgia **13**(1): 21-27.

Sandrini, G., I. Milanov, S. Malaguti, M. P. Nigrelli, A. Moglia and G. Nappi (2000). "Effects of hypnosis on diffuse noxious inhibitory controls." Physiol Behav **69**(3): 295-300.

Shahani, B. T. and R. R. Young (1971). "Human flexor reflexes." J Neurol Neurosurg Psychiatry **34**(5): 616-627.

Szlavik, R. B. and H. de Bruin (1999). "The effect of stimulus current pulse width on nerve fiber size recruitment patterns." Med Eng Phys **21**(6-7): 507-515.

Toledo, T. A., C. N. Vore, F. A. Huber and J. L. Rhudy (2024). "The effect of emotion regulation on the emotional modulation of pain and nociceptive flexion reflex." Pain **165**(6): 1266-1277.

Wesselink, W. A., J. Holsheimer and H. B. Boom (1999). "A model of the electrical behaviour of myelinated sensory nerve fibres based on human data." Med Biol Eng Comput **37**(2): 228-235.

Willer, J. C. (1980). "Anticipation of pain-produced stress: electrophysiological study in man." Physiol Behav **25**(1): 49-51.

Willer, J. C., F. Boureau and D. Albe-Fessard (1979). "Supraspinal influences on nociceptive flexion reflex and pain sensation in man." Brain Res **179**(1): 61-68.

Ydrefors, J., T. Karlsson, U. Wentzel Olausson, B. Ghafouri, A. C. Johansson, H. Olausson, B. Gerdle and S. S. Nagi (2020). "Automated Nociceptive Withdrawal Reflex Measurements Reveal Normal Reflex Thresholds and Augmented Pain Ratings in Patients with Fibromyalgia." J Clin Med **9**(6).
